# An Analysis of Primary Medical Care-An International Study

**Published:** 1980

**Authors:** 


### AN ANALYSIS OF PRIMARY MEDICAL CARE – AN INTERNATIONAL STUDY

**W. J. Stephen**

**£20.00**

This book is difficult to review and evaluate for the simple reason that it is unique. It is the only book that I know of which attempts to compare and contrast Primary Medical Care in a wide variety of countries. Dr. Stephen, a GP in Wells, has travelled widely to gather information, but also relies on information and statistics gathered from a variety of other sources. He examines a total of 23 different countries, mostly so-called developed countries of both East and West, but also includes a chapter on the developing countries. For each country there is a brief study of the historical and geographical factors which have influenced the development of Primary Medical Care, a short section on Finance and Medical Insurance, a description of the present system of Primary Care both Urban and Rural, and often short sections covering Maternity, Child Care, Emergency Care, the role of the Hospitals, and the development of Medical Education. Perhaps the most interesting section of each chapter is the author's personal assessment, especially of those countries which he has personally visited.

The book has very obvious limitations. Its scope is enormous and obviously one author cannot keep up-to-date with a continuously changing process over such a wide area. The conclusions obviously reflect the author's personal view of Primary Care. However, the fact that one man has undertaken this project is in itself a remarkable achievement.

Perhaps the main question the book asks is how best can any Government allocate its limited resources. Sometimes one reads criticism of inadequate facilities, while in other countries expensive equipment is readily accessible but under-used. The same of course can be said for the deployment of medical personnel.

When reading this book one is naturally comparing other systems to our own. Anyone interested in the future of General Practice, either on an individual basis or on a National scale, can learn a lot from this study. However, it is in the sphere of General Practice in this country that the book has its greatest failings. Here the description of General Practice is based largely on the early 1970's. We have seen many changes since then including re-organisation with increasing involvement of general practitioners in Management, improvement in undergraduate and postgraduate education, in particular vocational training. We have seen the development of more purpose-built premises with better equipment, and we have also been blessed with improved access to hospital facilities.

Paradoxically this criticism only underlines the value of the book. Primary Care is changing and there is plenty of scope for improvement, and we can all learn a great deal from studying the way it is practised in other countries. This book should make very interesting reading for anyone concerned with our Health Service.

